# Advances in clinical trial design for development of new TB treatments: A call for innovation

**DOI:** 10.1371/journal.pmed.1002769

**Published:** 2019-03-22

**Authors:** Christian Lienhardt, Payam Nahid

**Affiliations:** 1 Unité Mixte Internationale TransVIHMI (UMI 233 IRD–U1175 INSERM, Université de Montpellier), Institut de Recherche pour le Développement (IRD), Montpellier, France; 2 Division of Pulmonary and Critical Care Medicine, University of California San Francisco, San Francisco, California, United States of America

## Abstract

Christian Lienhardt and Payam Nahid launch the Collection on Advances in Clinical Trial Design for Development of New Tuberculosis Treatments.

After decades of stagnation, research in tuberculosis (TB) therapeutics is experiencing a renaissance, with an increasing number of new and repurposed compounds undergoing evaluation as part of novel treatment regimens. This is much welcome progress, since current regimens are not ideal due to the long duration of treatment required, toxicities, drug–drug interactions, and high costs—particularly for treatment of the various forms of drug-resistant TB (DR-TB). The development of new TB drugs is, however, complex, lengthy, and costly [1], and the pathway to proven new TB treatment regimens is fraught with numerous obstacles and uncertainties [2]. In this *PLOS Medicine* Collection, “Advances in Clinical Trial Design for Development of New Tuberculosis Treatments,” we highlight key obstacles and identify potential solutions that will help avoid misadventures and in turn maximize the likelihood of success in identifying new drugs and regimens through a rejuvenated global interest in TB therapeutics. With the emergence of several new chemical entities expected to transition into clinical testing in the next 5 years, the possibility of ultrashort (i.e., requiring treatment for weeks rather than months) regimens for active TB is no longer fanciful. Investigators in the field have learned much from recent TB clinical studies, and we anticipate that well-designed and conducted clinical trials evaluating the next generation of drugs and regimens will, with some good fortune, lead to identification of the ultrashort, safe, and effective regimens so desperately needed.

Treatment of TB relies on a synergistic combination of drugs (traditionally categorized as bactericidal or sterilizing) administered for sufficient time to achieve definitive nonrelapsing cure and to prevent selection of drug-resistant mutants [3]. The treatment of drug-susceptible TB (DS-TB) is well codified, with a standard combination of 4 drugs given for a duration of 6 months [4]. This regimen is the result of a series of clinical studies conducted in several countries, which demonstrated the efficacy of short-course regimens of 6–8 months’ duration in patients with pulmonary disease [5]. These trials played a key role in the establishment of short-course chemotherapy worldwide, allowing treatment of DS-TB to be based on the best available evidence [6]. Since then, clinical trials and programmatic experience have shown that the standard 6-month isoniazid/rifampicin-based regimen, when adhered to, performs consistently well in a wide variety of settings and can serve as a reliable control regimen against which investigational regimens can be compared [4]. The situation is, however, more complicated for DR-TB. In the absence of controlled trials comparing different regimens to a recognized “gold standard” treatment, the current recommendations for therapy rely on early-phase culture-conversion results, observational studies, and a few late-phase clinical trials [7]. The number and type of drugs required to treat patients with DR-TB has long been a matter of debate and controversy despite agreement on basic principles such as the minimum number of drugs to use and minimum duration of treatment. As a result, the efficacy of recommended DR-TB treatment regimens has been shown to vary widely in clinical studies and programs [8,9].

The need for solid evidence from randomized controlled trials has led the TB research community to adopt a design widely used in HIV research for the development of new antiretrovirals, in which patients are randomized to receive either a new drug or placebo in addition to a defined “optimized background regimen,” usually the best available standard of care [10]. This approach has been used in the development of bedaquiline [11] and delamanid [12], the first two new drugs approved for TB treatment since the late 1980s. While this research design assesses the added value, if any, of a given investigational drug, the approach leaves unresolved the question of the optimal drug combination in which to include the new agent [13]. As a result, additional clinical trials are then needed to identify the best options for treatment using new drugs in variable combinations, resulting in additional years of delay in producing the best evidence for global policy-making decisions. In parallel, practical recommendations are needed for the use of any newly approved drugs, along with guidance for countries and programs as to which combinations are safe, tolerable, and efficacious, an endeavor that requires systematic reviews and meta-analyses of observational cohort studies and programmatic data, which carry significant limitations. This approach is not sustainable, practical, or efficient and raises the need for a shift to a more efficient and seamless development process that allows the testing of novel treatment regimens, including one or more promising new or repurposed medicines, early in the clinical development pathway. Some stakeholders, such as the TB Alliance, therefore proposed a “unified approach to TB regimen development” addressing the joint development of new drugs and regimens for both DS-TB and DR-TB [14]. Also, the International Union against Tuberculosis and Lung Diseases opted to investigate the safety and efficacy of a set combination regimen of 9–12-months’ duration for the treatment of DR-TB in parallel through a randomized controlled trial [15] and observational studies undertaken within programmatic research conditions [16]. However, the availability of results from these various studies at different points in time and questions arising from the challenge of interpreting and integrating data from various methodologies were found to limit the adequacy of these complementary approaches for development of therapies [17].

Duly concerned with the need to base its normative treatment recommendation on the best available evidence [18], and to produce guidelines that would be readily usable in daily practice in all settings, WHO opted to establish minimal and optimal benchmarks for TB regimen development using industry-accepted target product profile (TPP) principles [19]. These TPPs for new anti-TB regimens, referred to as “target regimen profiles” (TRPs), describe the minimum and optimal attributes and characteristics of future TB regimens to guide the development process [20]. A population-level modeling analysis evaluating the potential impact of various regimen characteristics on the TB epidemic highlighted the paramount importance of regimen efficacy to exert the largest impact on reduction of TB cases and deaths, both for DS-TB and DR-TB [21]. Other characteristics such as shorter duration of, or increased adherence to, treatment were shown to have important effects by enabling more people with TB to receive appropriate and timely therapy. Most importantly, this model highlighted the difficulty of improving all potential characteristics simultaneously in a single regimen, leading developers to consider weighing in inevitable trade-offs (e.g., higher cure rates may be difficult to achieve simultaneously with shorter treatment duration, and simpler or better-tolerated regimens may be less robust to emergence of drug resistance) that are duly addressed in the TRPs.

Given the recommended regimen characteristics, the implementation of TRPs stimulated the question of which clinical trial designs and features should be optimally used for the development of new anti-TB regimens. Major challenges exist along the current lengthy development pathway [1], including the lack of direct indicators of treatment response, the lack of reliable surrogate markers of treatment outcomes, and the lack of predictive quantitative relationships between Phase II and Phase III outcomes [22]. To accelerate and streamline the development of new TB regimens, the therapeutics research community needs to establish clear and rationally justified approaches for the choice of drug combinations, trial design, selection of endpoints, and analysis [23,24], taking into account new developments in individual drugs’ pharmacokinetic and pharmacodynamic characteristics, microbiological aspects, use of biomarkers, standardization of approaches and data collection, as well as drug effects in key patient populations.

From the regimen developer’s perspective, it is apparent that a new treatment regimen must bring a value proposition, beyond efficacy or safety targets. Products with broader applications (e.g., for eligible populations) gain in terms of delivery and scalability/distribution or cost and can bring substantial impact and value that define the developmental pathway. Sponsors and donors should evaluate the needs of the market and develop programs based on those needs. In conjunction, decisions about progress from Phase II to Phase III studies continue to involve significant uncertainty, and these limitations need to be considered when designing Phase III trials. Also, the issue of the control groups most appropriate for a given trial situation needs careful consideration. It thus appears that each development program needs to determine the most appropriate approach to trial design, depending on the situation and the questions to be addressed.

From both programmatic and patient perspectives, the recent pooled individual patient-level analysis of three treatment-shortening trials examining the efficacy and safety of 4-month combination regimens, including third-generation fluoroquinolones for the treatment of DS-TB [25–27], provided critically important insights relevant to TB treatment in the field and to therapeutics research [28]. Whereas these trials independently failed to show noninferiority of the 4-month experimental regimens tested, as compared to the 6-month control regimen, 80% of patients were cured. The pooled analysis of these trials found that patients with minimal disease, defined as low bacterial burden or absence of lung cavities, would be eligible for 4-month treatments [28]. Conversely, patients with high baseline smear, cavitation on chest X-ray, HIV coinfection, and low body mass index defined hard-to-treat phenotypes that would need more than the standard 6-month treatment duration to achieve the highest possible cure rates. In addition, even minimal nonadherence (i.e., missing 1 in 10 doses) to the current standard regimen was found to be a significant risk factor for unfavorable outcome, independent of treatment duration. These findings provide a strong evidence-based framework for investigating different approaches to achieving better patient-oriented treatment—such as the stratified medicine approach—and emphasize the importance of maximizing adherence in clinical trials and in real-world conditions.

These issues illustrate the need for obtaining maximally informative and reliable data from controlled trials, as these are paramount for the development of policy for wide public health use and for guideline development. To address these coherently, in March 14–16, 2018, WHO organized a technical consultation on “Advances in Clinical Trial Design for New TB Treatments” to identify and outline, through expert consensus, the optimal characteristics of clinical trial designs to inform policy guidance for the development of new TB regimens. Building on the lessons learned from the rich history of TB clinical trials, the WHO technical consultation [29] reviewed the various research designs and tools currently used in the conduct of clinical trials for development of new TB treatments and made a series of proposals to advance these further, seeking to move from evolutionary change informed by history to a bolder approach to innovation geared to the future. These are the aims of this *PLOS Medicine* Collection, which we are launching on World TB Day 2019, beginning with the accompanying paper from Patrick Phillips and colleagues [30] on the changing landscape of clinical trial design for development of TB therapeutics. Further articles will be added to the Collection in due course, and the Collection will be available in its entirety alongside this paper once all the articles have been published.

## Abbreviations

DR-TB: drug-resistant TB
DS-TB: drug-susceptible TB
TB: tuberculosis
TPP: target product profile
TRP: target regimen profile
